# Erroneously Suspected Ovarian Cancer in a 38-Year-Old Woman with Pelvic Inflammatory Disease and Chlamydia

**DOI:** 10.1155/2017/2514613

**Published:** 2017-12-13

**Authors:** Romana Brun, Juliane Hutmacher, Daniel Fink, Patrick Imesch

**Affiliations:** Division of Gynecology, University Hospital of Zurich, Zurich, Switzerland

## Abstract

Chlamydia trachomatis is the most common bacterial cause of sexually transmitted disease and can cause pelvic inflammatory disease (PID), leading to severe outcomes such as ectopic pregnancy, infertility, or pelvic pain. We report a case of a 38-year-old patient with abdominal pain and dyspareunia. Clinical examination revealed diffuse abdominal tenderness. Vaginal and abdominal sonography showed substantial ascites and CA-125 level was elevated. Therefore, the attendant physician performed an abdominal CT scan for further diagnosis. Radiographically diffuse peritoneal enhancement, consistent with peritoneal carcinomatosis, 4-quadrant ascites, and slightly enlarged ovaries with solid and cystic structures were diagnosed, leading to the suspicion of ovarian cancer. In addition, the results of the cervical smear PCR for chlamydia were positive. Due to the positive chlamydia result, the suspicious CT scan, and the young age, we decided to perform a diagnostic laparoscopy as a first step. Intraoperatively, the ovaries were of normal aspect without any cancerous lesions. However, the ascites and the yellow-reddish jelly-like deposits were consistent with acute PID. Thus, chlamydia infection may simulate the presentation of ovarian cancer. Therefore, especially in young patients, we recommend careful scrutiny of every diagnosis of ovarian cancer even if its presentation seems to be typical.

## 1. Introduction

Chlamydia trachomatis is an obligate intracellular bacterium and is the most common bacterial cause of sexually transmitted genital infections [1, 2]. Approximately 100 million new cases of genital infections are diagnosed worldwide every year [1]. Infected people are frequently asymptomatic and are not aware of their infection. Urogenital chlamydial infection can cause pelvic inflammatory disease (PID), which can lead to severe outcomes such as ectopic pregnancy or infertility, chronic pelvic pain, preterm birth, fetal growth retardation, and low birth weight as well as perinatal diseases such as conjunctivitis, pneumonia, and infant death [1, 3, 4]. The prevalence of chlamydial genital infections is estimated to be similar in women and men, with the ranges 3–5.3% and 2.4–7.3%, respectively [1, 4]. Chlamydia trachomatis is transmitted by direct mucosal contact between two individuals during sexual intercourse (vaginal, anal, or oral sex) or at birth through an infected cervical canal [1].

## 2. Clinical Presentation

**Figure 1:**
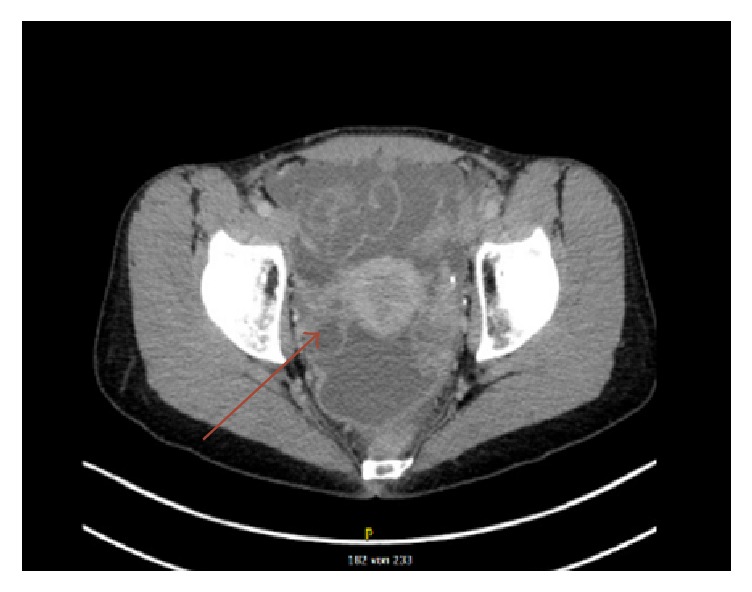
CT scan showing diffuse peritoneal enhancement consistent with peritoneal carcinomatosis, 4-quadrant ascites, and slightly enlarged ovaries with solid and cystic structures. The arrow refers to the right ovary.

A 38-year-old female patient was admitted to the Department of Gynecology, University Hospital Zurich, with suspicion of ovarian cancer. Prior to admission, she had complained of abdominal pain and dyspareunia and had consulted her private gynecologist. A clinical examination revealed positive bowel sounds over all 4 quadrants and diffuse abdominal tenderness. The past medical history was unremarkable. The patient showed no signs of peritonism and no typical tenderness of the cervix. Vaginal sonography revealed a moderate amount of ascites in the pouch of Douglas. Serum tumor marker cancer antigen CA-125 was significantly elevated with 482 U/ml, suggestive of an underlying malignancy. The patient was admitted to a radiologist and scheduled for an abdominal ultrasound. The abdominal ultrasound showed 4-quadrant ascites, and for further diagnosis an abdominal CT scan was performed. In the CT scan, diffuse peritoneal enhancement, suspicious of peritoneal carcinomatosis, 4-quadrant ascites, and slightly augmented ovaries with solid and cystic structures became apparent (Figure 1). No pathological lymph nodes could be detected.

On admission, the patient presented in relatively good general condition, afebrile with normal cardiovascular parameters. Laboratory studies were within normal ranges for hemoglobin (118 g/l) and leucocytes (7.33 G/l). Thrombocytes (446 G/l) and CRP (16 mg/l) showed mildly increased levels. Regarding the tumor markers, an increased CA-125 (612 kU/l) was observed, with normal ranges for CA 15-3 (23.5 kU/l), CA 19-9 (8.4 kU/l), and CEA (1.6 *μ*g/l). A cervical smear PCR for chlamydia showed a positive result.

As a first step during the hospitalization, the patient and her partner were treated with Azithromycin 1 g for 2 days prior to surgery.

**Figure 2:**
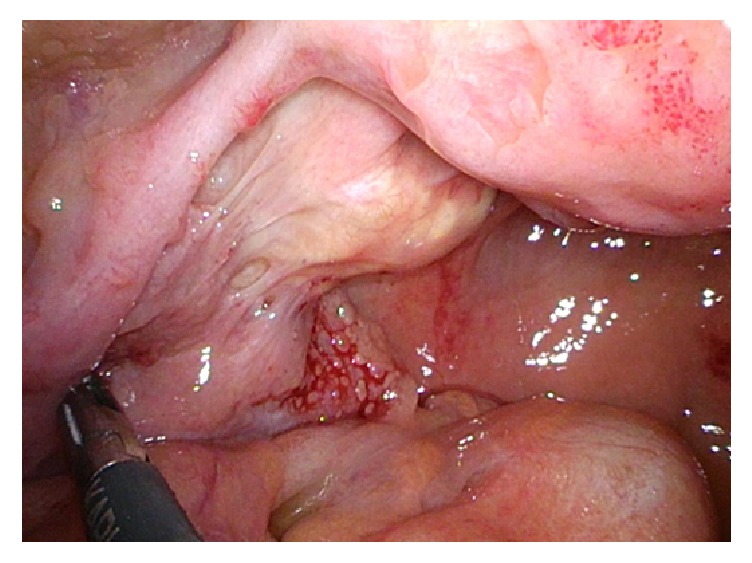
Left ovary and Fallopian tubes with reddish-yellow deposits.

A diagnostic laparoscopy was performed as a next step. Exploration of the abdominal cavity revealed an important amount of brown, dull liquid. There were multiple yellow-reddish jelly-like deposits on the Fallopian tubes, ovaries, Douglas pouch, and uterus and also on the peritoneum. The intestines also presented with a slightly reddish-yellowish marble-like coloring. The peritoneum showed no whitish, cancer-like deposits (Figure 2). The ovaries were slightly adherent to the pelvic walls, but, otherwise, of normal aspect without any cancerous lesion. The entire intra-abdominal organs were very sensitive and easily bloody after contact.

**Figure 3:**
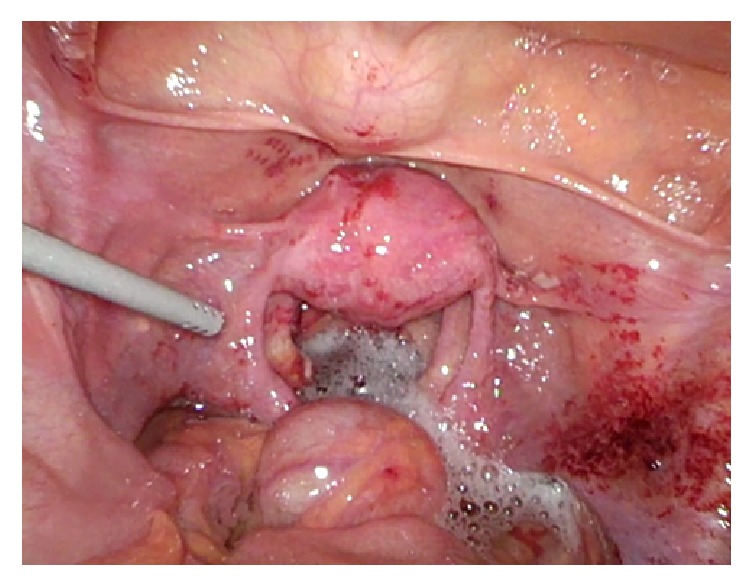
Foaming lavage, easily bloody after contact.

An ascites lavage was performed and sent for cytological analysis. Then, an intraoperative smear for general bacteriological analysis as well as a PCR smear for chlamydia and gonococcus was taken. Some biopsies of the deposits on the peritoneal wall near the liver, the Fallopian tubes, the Douglas and the pelvic peritoneum were also taken. At the end, we carried out an abdominal lavage with several liters of NaCl, during which an interesting reaction of the water in contact with the peritoneal walls was observed: it foamed (Figure 3).

The postoperative course was trouble-free except for slight abdominal pain. The patient remained afebrile during hospitalization. We treated the patient with selective antibiotics with doxycycline because of the positive chlamydia diagnosis before surgery and the absent inflammatory blood parameters. The PCR result was positive for chlamydia; hence we continued monotherapy with doxycycline 200 mg/day, initially intravenously for 3 days and after that per os for overall 14 days. The patient was discharged on the second postoperative day in good general condition.

**Figure 4:**
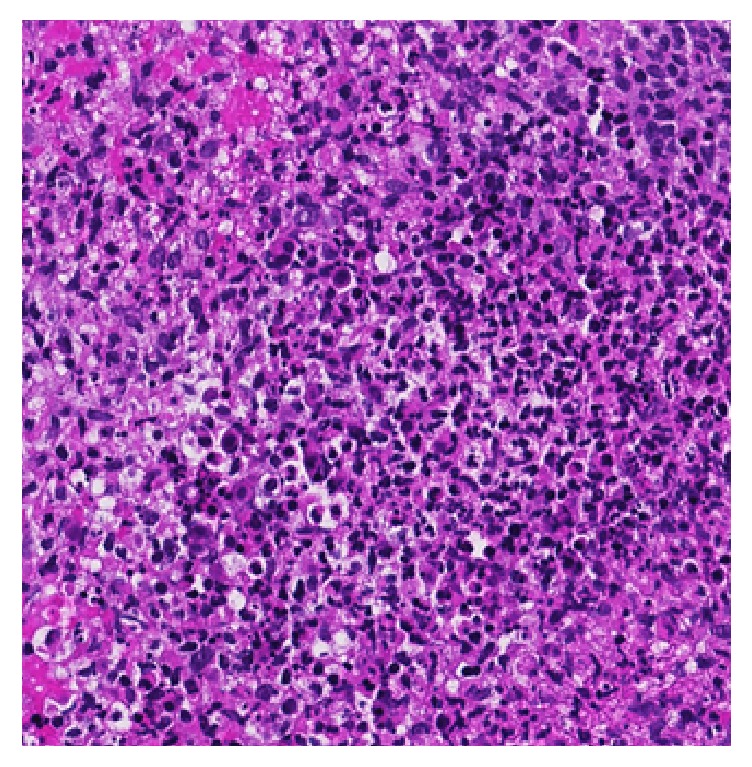
Histology: chronic-active inflammation with many plasma cells and eosinophilic granulocytes.

The pathology report disclosed no malignancy, but cytology of the lavage presented a high rate of lymphocytes and histiocytes as well as few neutrophil granulocytes. The biopsies showed a chronic-active inflammation with numerous plasma cells and eosinophilic granulocytes (Figure 4).

A follow-up 6 days after surgery showed decreased ascites. The patient complained of very slight abdominal pain, showed no signs of inflammation or fever, and presented herself in good general health condition with well-being. The chlamydia smear PCR control will be done in 6 weeks by her attending gynecologist.

## 3. Discussion

Since radiological interpretation and an elevated tumor marker can be suspicious of cancer, we must be aware of early interpretations.

Sran et al. presented a case of a 17-year-old girl with abdominal pain, diffuse peritoneal tenderness, ascites, and mildly elevated CA-125 which, after a first misleading diagnosis of ovarian cancer, appeared to be a PID caused by chlamydia [5]. Ugianskiene described another case of ascites and adnexal mass that simulated a malignant neoplasm [6]. In this case, laparotomy was performed and the biopsies taken during surgery showed chronic lymphofollicular inflammation, suggesting a chlamydia infection, which was treated with antibiotics.

According to our case, we recommend careful scrutiny of every diagnosis of ovarian cancer, especially in young patients, even if its presentation seems to be typical. Chlamydia infection can simulate ovarian cancer. It should be noted that a radiological interpretation of an enhanced peritoneum as peritoneal carcinomatosis has to be questioned. Even in cases of ascites in combination with an elevated cancer antigen, we postulate the performance of a laparoscopy with biopsies and cytology as a first step, to confirm any diagnosis before a laparotomy is done.
